# Exome sequencing characterizes the somatic mutation spectrum of early serrated lesions in a patient with serrated polyposis syndrome (SPS)

**DOI:** 10.1186/s13053-017-0082-9

**Published:** 2017-11-29

**Authors:** Sukanya Horpaopan, Jutta Kirfel, Sophia Peters, Michael Kloth, Robert Hüneburg, Janine Altmüller, Dmitriy Drichel, Margarete Odenthal, Glen Kristiansen, Christian Strassburg, Jacob Nattermann, Per Hoffmann, Peter Nürnberg, Reinhard Büttner, Holger Thiele, Philip Kahl, Isabel Spier, Stefan Aretz

**Affiliations:** 1 Institute of Human Genetics, University of Bonn, Bonn, Germany; 2 Department of Anatomy, Faculty of Medical Science, Naresuan University, Phitsanulok, Thailand; 3 Center of Excellence in Medical Biotechnology, Naresuan University, Phitsanulok, Thailand; 4 Institute of Pathology, University of Bonn, Bonn, Germany; 5 Center for Hereditary Tumor Syndromes, University Hospital Bonn, Bonn, Germany; 6 Institute of Pathology, University of Cologne, Cologne, Germany; 7 Department of Internal Medicine I, University of Bonn, Bonn, Germany; 8 Cologne Center for Genomics (CCG), University of Cologne, Cologne, Germany; 9 Center for Molecular Medicine Cologne, University of Cologne, Cologne, Germany; 10 Department of Genomics, Life & Brain Center, University of Bonn, Bonn, Germany; 11 Institute of Medical Genetics and Pathology, University Hospital Basel and Department of Biomedicine, University of Basel, Basel, Switzerland; 12 Heinz-Werner-Seifert-Institut für Dermatopathologie Bonn, Bonn, Germany; 13 Institute of Human Genetics, Center for Hereditary Tumor Syndromes, University of Bonn, Sigmund-Freud-Str. 25, D-53127 Bonn, Germany

**Keywords:** Familial colorectal cancer (CRC), Exome sequencing, Hyperplastic polyposis syndrome (HPS), Serrated polyposis syndrome (SPS), Serrated pathway, Somatic mutations

## Abstract

**Background:**

Serrated or Hyperplastic Polyposis Syndrome (SPS, HPS) is a yet poorly defined colorectal cancer (CRC) predisposition characterised by the occurrence of multiple and/or large serrated polyps throughout the colon. A serrated polyp-CRC sequence (serrated pathway) of CRC formation has been postulated, however, to date only few molecular signatures of serrated neoplasia (*BRAF*, *KRAS, RNF43* mutations, CpG Island Methylation, MSI) have been described in a subset of SPS patients and neither the etiology of the syndrome nor the distinct genetic alterations during tumorigenesis have been identified.

**Methods:**

To identify somatic point mutations in potential novel candidate genes of SPS-associated lesions and the involved pathways we performed exome sequencing of eleven early serrated polyps obtained from a 41 year-old female patient with clinically confirmed SPS. For data filtering and analysis, standard pipelines were used. Somatic mutations were identified by comparison with leukocyte DNA and were validated by Sanger sequencing.

**Results:**

The *BRAF* p.V600E or *KRAS* p.G12D mutation was identified in six polyps (~50%) and not found in polyps from the distal colon. In addition, we found seven unique rare somatic alterations of seven different genes in four serrated tumours, all of which are missense variants. The variant in *ABI3BP* and *CATSPERB* are predicted to be deleterious*.* No established cancer gene or candidate genes related to serrated tumorigenesis were affected.

**Conclusions:**

Somatic mutations seem to be rare events in early hyperplastic and serrated lesions of SPS patients. Neither frequently affected genes nor enrichment of specific pathways were observed. Thus, other alterations such as non-coding variants or epigenetic changes might be the major driving force of tumour progression in SPS.

**Electronic supplementary material:**

The online version of this article (10.1186/s13053-017-0082-9) contains supplementary material, which is available to authorized users.

## Background

Serrated colorectal polyps represent a heterogeneous group of lesions that includes hyperplastic polyps (HPs), sessile serrated polyps/adenomas (SSP/A) with or without dysplasia, traditional serrated adenomas (TSA) and mixed polyps with a combination of different characteristics [1, 2].

HPs belong to the most common colorectal polyp types and were traditionally regarded as harmless, non-neoplastic lesions, however, there is growing evidence that at least some HPs have a malignant potential [3, 4]. According to this model, a small percentage of HPs –often large and right-sided – progress to other serrated polyps, in particular SSP/A, and then evolve to colorectal cancer (CRC). Commonly, HPs are smaller than 5 mm in diameter and tend to appear more frequently in the distal colon and rectum whereas SSA/Ps are found more often in the right than the left colon and are larger than 5 mm in diameter [5].

Nowadays, it is assumed that serrated polyps are part of an alternative (serrated) pathway of colorectal carcinogenesis. Similar to the classical adenoma-adenocarcinoma sequence [6], a serrated polyp-carcinoma sequence (serrated neoplasia pathway) was postulated where serrated polyps replace the traditional adenoma as the precursor lesion to CRC [7]. It is assumed, that serrated adenocarcinomas, which represent approximately 10% of sporadic CRC, arise via the serrated polyp-carcinoma sequence.

While the step-wise progression of premalignant lesions to carcinomas in the classical adenoma-carcinoma sequence is characterised by chromosomal instability (CIN) and *APC* or *KRAS* mutations, the molecular profiles described in serrated neoplasias include specific *BRAF* and *KRAS* mutations, microsatellite instability (MSI), and a CpG island methylator phenotype (CIMP) [8–11]. Nonetheless, although the serrated pathway is regarded to be the second most important pathway leading to CRC, the molecular steps of tumorigenesis are still largely unknown. Previous studies identified two potential RNA markers for SSP/As (*ANXA10*, *CLDN1*) by microarray analyses [12, 13] and somatic mutations in established cancer pathways [14]. Very recently, a unique signature of differentially expressed genes was found in early SSP/As by a comprehensive RNA-Seq analysis that discriminates between SSP/As and HPs [15]. Interestingly, an almost complete gene overlap between sporadic and syndromic SSP/As was observed.

Hyperplastic polyposis syndrome (HPS), now mostly designated as serrated polyposis syndrome (SPS), is a yet clinically defined colorectal polyposis characterised by the presence of multiple and/or large serrated polyps throughout the large intestine. In this way, SPS can be regarded as a model disease of the serrated neoplasia pathway [11]. The empirical WHO diagnostic criteria require 1) at least 5 serrated polyps proximal to the sigmoid colon, with two or more >10 mm, or 2) any number of serrated polyps proximal to the sigmoid colon with an affected 1° relative, or 3) >20 serrated polyps of any size throughout the colon [1].

If left untreated, affected persons and their relatives have an increased lifetime risk for the development of CRC. Therefore, SPS patients undergo endoscopic surveillance with removal of polyps or surgical colonic resection. The prognosis of these early-manifesting lesions can be decisively improved through the timely detection of the tumour by established disease specific surveillance programs, however, the optimal extent and frequency of surveillance is unknown so far. Interestingly, CRC even occurred in patients despite all SSP/As were excised [16].

In contrast to many other inherited gastrointestinal polyposis syndromes, the etiology of SPS remains unclear. Although SPS was initially considered to be non-inherited, familial clustering and a high risk (up to 50%) of CRC in first degree relatives of SPS patients has been described [17]. Based on these reports, a yet unidentified underlying genetic defect seems to play a significant role in SPS, however, the possible modes of inheritance are still unknown. A previous study proposed germline mutations in oncogene-induced senescence pathways as predisposing factor [18]. In addition, both truncating somatic and germline mutations of *RNF43* were found in SSA and a few patients with (autosomal dominant) SPS, respectively [18, 19].

The characteristic molecular profile of serrated neoplasia mentioned above was described in a subset of SPS-associated polyps. Probably, the current definition of SPS subsumes several clinical subtypes with different cancer risks and prognosis, however, in the absence of valid biomarkers no distinction between those subtypes is possible yet.

As the etiology of the syndrome and the distinct genetic alterations during tumorigenesis are still poorly understood, this study aimed to characterise the spectrum of somatic mutations in protein-coding genes present in early serrated lesions from different colonic parts of a single SPS patient.

## Methods

### Patient / data collection

The female patient presented at 38 years of age due to unclear abdominal trouble. A colonoscopy resulted in the diagnosis of a colorectal polyposis. Initially, several flat, broad-based polyps resembling a dense lawn were found within the caecum in addition to at least 10 more, partly flat, partly pedunculated polyps with a diameter of up to 20 mm. Four more colonoscopies were performed; altogether more than 45 polyps covering the whole colon were identified and partly removed. Around 20 polyps were histologically examined, the vast majority was classified as hyperplastic polyp and a few as serrated polyp, all without severe dysplasia. Neither typical adenomatous polyps nor carcinomas were found. A gastroduodenoscopy showed normal results. The WHO diagnostic criteria for SPS were met by the patient. A *MUTYH*-associated polyposis (MAP) was excluded by screening of the *MUTYH* gene as described elsewhere [20].

At the age of 41 years, a subtotal colectomy with ileoanal anastomosis was performed. The removed colon was examined carefully by an experienced pathologist from the Institute of Pathology in Cologne, the reference pathology of the German HNPCC consortium. Overall, 21 polyps up to 18 mm were seen, removed, and immediately fresh-frozen; all of them were classified as hyperplastic or sessile serrated polyps later on.

Beside of this, the patient had an inconspicuous medical history without important pre-existing disease. The family history was unremarkable, in particular regarding gastrointestinal polyps, extraintestinal tumours, or cancer, however, the father and his three half siblings had no colonoscopy.

The study was approved by the local ethics review board (Medical Faculty of the University of Bonn ethics review board no. 208/12), and a written informed consent was obtained from the patient.

### DNA extraction from blood and tissue samples

Leukocyte-derived genomic DNA was isolated from peripheral blood with standard protocols as described elsewhere [21]. DNA from macrodissected, fresh-frozen serrated polyp tissue was extracted using the proteinase K treatment followed phenol extraction as described elsewhere [22].

### Targeted genetic profiling of polyps

The targeted mutation screening was performed with the commercial kit TruSight®Tumor 15 Sequencing Panel (Illumina, San Diego) which includes 15 established cancer-associated genes (*AKT1, BRAF, EGFR, ERBB2, FOXL2, GNA11, GNAQ, KIT, KRAS, MET, NRAS, PDGFRA, PIK3CA, RET*, *TP53).* DNA concentration was determined with the Quanti Fluor dsDNA Systems Assay Kit (Promega, Mannheim, Germany) on the Quantus Fluorometer (Promega). Twenty nanogram DNA was used for library preparation. Target enrichment and high-throughput sequencing was performed according to the manufacturers protocol. All samples were sequenced on an Illumina MiSeq sequencer (Illumina, San Diego, CA). Pooled libraries (5 ng/μl) were spiked with 1% PhiX DNA (Illumina) and paired-end sequenced was performed with the “MiSeq reagent Kit V3 (600-cycles)” (Illumina). FastQ files generated by the MiSeq Reporter were used as data output. All variants identified were confirmed by high resolution melting (HRM) analysis and by Sanger sequencing as previously reported [23].

### Microsatellite analysis

Microsatellite analysis was performed on matched tumour and normal DNA samples. This involved use of the National Cancer Institute (NCI) reference marker panel for the evaluation of microsatellite instability (MSI) in colorectal cancer. This panel consists of two mononucleotide (BAT25, BAT26), and three dinucleotide (D2S123, D5S346 and D17S250) repeats [24]. Tumour DNA was extracted from macrodissected tumour tissue. Normal DNA was extracted from peripheral blood leukocytes. Tumours were scored as highly unstable (MSI-H) if two or more of these five markers exhibited additional alleles, and as stable (MSS) if none of the five markers showed instability.

### Whole exome sequencing (WES)

For whole-exome sequencing, 1 μg of DNA was fragmented with sonication technology (Bioruptor, Diagenode). The fragments were end-repaired and adaptor-ligated, including incorporation of sample index barcodes. After size selection, a pool of all 6 libraries was subjected to an enrichment process with the SeqCap EZ Human Exome Library version 3.0 kit (Roche NimbleGen). The final libraries were sequenced on an Illumina HiSeq 2000 sequencing instrument (Illumina, San Diego, U.S.) with a paired-end 2 × 100 bp protocol.

### Exome analysis and filtering

Primary data were filtered using the Illumina Realtime Analysis (RTA) software version 1.8. Subsequently, the reads were mapped to the human genome reference build GRCh37 using the BWA alignment algorithm [25]. GATK version 1.6 [26] was used to mark duplicated reads, perform local realignment around short insertions and deletions, recalibrate the base quality scores and call SNVs and short Indels together with SAMtools version 0.1.18 and *Dindel* version 1.01 [27]. On average, 104.7 million reads were uniquely mapped. 80.9% of all targeted regions were covered >30 times, with a mean coverage of 89. Scripts developed in-house at the *Cologne Center for Genomics* were applied to detect protein changes, affected donor and acceptor splice sites, and overlaps with known variants. Acceptor and donor splice site mutations were analyzed using a Maximum Entropy model [28].

Variant filtering was performed with the VARBANK graphical user interface *(* *https://varbank.ccg.uni-koeln.de* *)*, comparison with public variant databases and subsequent manual check to select relevant, likely pathogenic somatic mutations affecting protein coding genes. Variants were included if they 1) are truncating variants (nonsense mutations, frameshift deletions/insertions, mutations located at exon-flanking, highly conserved intronic splice sites), or apparent missense mutations predicted to be pathogenic by in-silico prediction tools (see below); 2) are located at a region with a minimal total coverage (read depth) of at least 10×; 3) had a variant allele frequency of ^3^5% in tumour DNA; and 4) were not detected in corresponding matching normal tissue (leukocyte DNA).

Subsequently, all common variants which are reported with a minor allele frequency (MAF) of >1% in the germline according to population-based databases (NCBI dbSNP, ExAC, 1000Genomes) were removed since those variants are very likely to represent rare polymorphisms or low-penetrant variants rather than pathogenic somatic driver mutations of tumorigenesis. In addition, filtering was performed against an in-house database containing variants from 511 exomes from epilepsy patients in order to exclude pipeline-related artifacts or population-specific rare polymorphisms. The remaining variants were further checked by visual inspection using the VARBANK read browser to exclude obvious false positive variants / artifacts.

Afterwards, 123 genes which had been proposed as driver candidate genes of the serrated pathway due to genetic alterations or epigenetic silencing in serrated lesions, including established causative genes or candidate genes for (hereditary) colorectal tumours (Additional file 1: Table S1) [19, 29–36] were analyzed for the presence of any rare variant.

### Sanger sequencing

All rare variants in promising candidate genes were validated by Sanger sequencing of tumour DNA, assuming that relevant somatic mutations are present in an allelic fraction of ≥10%. DNA sequences were obtained from the UCSC Genome Browser (hg19). Primers were designed using Primer3 V.0.4.0 (*http://frodo.wi.mit.edu/primer3/input.htm*) (Additional file 1: Table S2). Stored genomic DNA was extracted from leukocytes and tumour DNA was used to amplify the coding regions and adjacent intronic sequences of the respective genes. PCR products were purified using the QIA quick PCR purification kit (Qiagen, Hilden, Germany), and sequenced on an ABI 3500xl Genetic Analyzer (Life Technologies) using the BigDye terminator kit version 1.1 (Life Technologies). The cDNA bases were numbered according to the gene reference sequence in GenBank, where 1 corresponds to the A of the ATG translation initiation codon.

### In-silico analysis

Splicing efficiencies of the normal and mutant sequences were calculated using the splice prediction program NNSPLICE 0.9 from BDGP (the Berkeley Drosophila Genome Project). Potentially deleterious effects of putative missense variants were predicted using the variant-based in-silico tools CADD (cutoff on deleteriousness: values >15) [37], PolyPhen-2, MutationTaster, and SIFT, as well as the functional gene constraint z- and %ExAC_RVIS-scores [38, 39]. For all rare interesting candidate variants, the allele frequencies in controls were again checked using the gnomAD [38], COSMIC, and TCGA databases.

## Results

Eleven representative polyps from all parts of the colon had been selected which cover a spectrum from very small (4 mm) to medium-sized (13 mm) lesions. All were classified as hyperplastic or sessile serrated polyps (Table 1)*.* All examined polyps demonstrated microsatellite stability (MSS) (data not shown).

**Table 1:** Results of histopathology and targeted molecular profiling of the eleven polyps

Tumour ID	Localization	Size (mm)	Histopathological feature	TruSight® Tumor 15 Sequencing	TruSight® mutant allele frequency (%)	Exome Sequencing	WES mutant allele frequency (%)	Validation by SS
T69	Caecum	5	hyperplastic polyp	*KRAS* p.G12D	21	*KRAS* p.G12D	8	confirmed
T70	ascending colon	7	hyperplastic polyp	*BRAF* p.V600E	26	*BRAF* p.V600E	17	confirmed
T71	ascending colon	7	hyperplastic polyp	*BRAF* p.V600E	28	*BRAF* p.V600E	12	confirmed
T72	ascending colon	8	hyperplastic polyp	*BRAF* p.V600E	17	*BRAF* p.V600E	14	confirmed
T73	ascending colon	6	hyperplastic polyp	–	–	–	–	–
T74	transverse colon	10	sessile serrated polyp	*BRAF* p.V600E	13	*BRAF* p.V600E	10	confirmed
T76	transverse colon	11	sessile serrated polyp	*BRAF* p.V600E	21	*BRAF* p.V600E	11	confirmed
T77	descending colon	8	hyperplastic polyp	–	–	–	–	–
T78	descending colon	4	hyperplastic polyp	–	–	–	–	–
T79	descending colon	4	hyperplastic polyp	–	–	–	–	–
T80	descending colon	13	hyperplastic polyp	–	–	–	–	–

*SS* sanger sequencing, *WES* whole exome sequencing

Prior to further filtering and validation steps of the exome sequencing data*,* we looked for variants in 123 genes including *RNF43* and other driver candidate genes of the serrated pathway and established causative genes or candidate genes for (hereditary) colorectal tumours. Thereby we identified the two common hotspot mutations *BRAF* V600E (c.1799 T > A;pVal600Glu; NM_004333.4) and *KRAS* G12D (c.35G > A;p.Gly12Asp; NM_033360.3) (8–17% of mutated reads) in 6/11 tumour samples (Table 1)*.* No further putative pathogenic variant could be identified in any of the remaining genes although the respective genomic regions had a minimum of 30× coverage in all polyp-derived DNA samples tested except for sample T69 which showed a lower coverage in general, and very few single exons of some genes with a coverage of <25× while *RNF43* was covered higher than 100× in all samples and exons.

Targeted genetic profiling of all polyps for 15 established cancer-associated genes (TruSight®Tumor15, Illumina) confirmed the *BRAF* V600E and *KRAS* G12D mutations in 5 (46%) and one (9%) polyps, respectively. No mutation was identified in the remaining 13 cancer-associated genes. All mutations could be further validated by Sanger sequencing (Additional file 2: Figure S1).

**Fig. 1:**
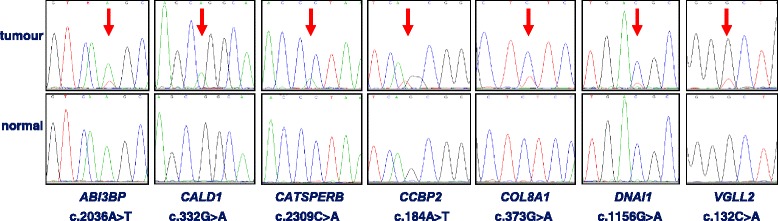
Sanger sequencing confirmed seven somatic missense mutations (upper row: tumour DNA; lower row: leukocyte DNA of the patient; for *COL8A1*, *DNAI1* and *VGLL2* reverse sequences are shown)

After applying the above mentioned filter criteria, the vast majority of variants was excluded. All of the remaining 25 unique variants were single base pair substitutions, the predominant mutation type (75%) were predicted missense variants caused by G > T transversions (Additional file 1: Table S2)*.* However, all but one (*CATSPERB*:c.2309C > A; p.Pro770His) of the G > T transversion could not be validated by Sanger sequencing, pointing to technical artifacts; in almost all of them the read depth was low (10-19×). Corresponding to the NGS results, seven somatic variants affecting seven genes were confirmed (both directions); all of them are predicted to be missense variants and all showed a low fraction of the variant allele indicating tissue heterogeneity / mosaicism in the tumour sample (Table 2, Fig. 1). All affected genes are expressed in colorectal tissue.

**Table 2:** Details of seven predicted missense variants which were validated by Sanger sequencing

Gene (NCBI Ref Seq)	Mutation	Tumour ID	Functions, pathways	SIFT / MT / PolyPhen-2	CADD^a^	z-Score^b^	%ExAC_RVIS^c^	TCGA / COSMIC	gnomAD	final assessment patho-genicity
*ABI3BP* (NM_015429.3)	c.2036A > T; p.K679 M	T71	prevention of tumorigenesis, proliferation of replicative senescence	D / DC / PD	24.6	−0.39	89.49	no report	no report	likely pathogenic
*CALD1* (NM_033140.3)	c.332G > A; p.R111Q	T69	potent repressor of cancer cell invasion; Schwann cell migration	D / P / N/A	26.2	0.24	62.76	no report	2.481e-5	VUS
*CATSPERB* (NM_024764.2)	c.2309C > A; p.P770H	T71	spermatogenesis, cell differentiation, multicellular organism development	D / DC / PD	24.6	0.93	4.00	no report	no report	likely pathogenic
*CCBP2* (NM_001296.4)	c.184A > T; p.S62C	T72	G-protein coupled receptor signaling pathway	T / P / B	0.006	−0.32	53.24	no report	no report	likely benign
*COL8A1* NM_020351.3)	c.373G > A; p.E125K	T71	migration and proliferation of vascular smooth muscle cells	T / DC / B	13.1	0.59	10,36	no report	no report	VUS
*DNAI1* (NM_012144.2)	c.1156G > A; p.V386I	T71	epithelial cilium movement, cell projection organization	T / P / B	11.3	−0.22	68.31	no report / 1 report^d^	2.832e-5	likely benign
*VGLL2* (NM_182645.2)	c.132C > A; p.S44R	T70	muscle differentiation and development of skeletal muscles	T / DC / PD	22.4	1.47	67.74	no report	no report	VUS

*B* benign, *D* damaging, *DC* disease causing, *MT* MutationTaster, *N/A* not applicable, *P* polymorphism, *PD* probably damaging, *T* tolerated, *VUS* variant of uncertain significance; ^a^cutoff on deleteriousness >15; ^b^positive Z scores indicate increased constraint (intolerance to variation), negative score implied that the gene shows more variants than expected; ^c^ values represent percentiles of %ExAC_RVIS scores. High values refer to tolerant genes while low values refer to intolerant genes; ^d^ in liver hepatocellular carcinoma

Each variant was present in a single polyp only. Four mutations occurred in the same polyp (T71), the other three were identified each in another polyp (T69, T70, and T72). No known cancer-associated gene was among the seven candidates. Data mining according to gene function and pathways demonstrated that the majority is involved in processes relevant for tumorigenesis such as cell differentiation and proliferation (Table 2). Most of the variants are neither reported as somatic, tumour-related variant in TCGA or COSMIC, nor as germline variant in large control cohorts (ExAC database) so far. Just the variants in *DNAI1* and *CALD1* have been reported in ExAC very rarely (heterozygote frequency < 0.01%); the *DNAI1* variant was found in a hepatocellular carcinoma (COSMIC) [40]. The *ABI3BP and CATSPERB* variants are predicted to be damaging by all three in-silico tools (Table 2).

## Discussion

In recent years, a characteristic molecular pattern was identified in both sporadic and SPS-associated serrated lesions, namely specific activating somatic mutations in two members of the mitogen-activated protein kinase (MAPK) cascade (*BRAF* and *KRAS*), a CpG Island Methylator Phenotype (CIMP), microsatellite instability (MSI) (reviewed in [7]), and a unique expression signature [15]. The frequency of specific alterations strongly depends on the number, location (right versus left colon) and histologic subtype of polyps examined. In addition, truncating mutations of the tumor suppressor gene *RNF43*, a transmembrane E3 ubiquitin protein ligase which acts as a Wnt signaling inhibitor by targeting the Frizzled receptor for degradation, was identified in sporadic SSP/A and TSA and as germline mutation in a small subset of SPS families [18, 19, 41].

Our results of exome sequencing, targeted genetic profiling and microsatellite analysis are consistent with published literature: the *BRAF* missense mutation V600E was found in 67–88% of HPs and 61–83% of SSP/A; the most common *KRAS* missense mutations (G12D, G12 V, G13D) in 6–17% of HP and in 7–25% of SSP/A. A CIMP, which might result in *MLH1* or *MGMT* (O6-methylguanine-DNA methyltransferase) silencing, was observed in 41–73% of HPs and 44–77% of SSP/A, respectively [30]. In contrast, MSI was rarely examined in serrated polyps and then usually not found in HPs and SSP/A except for a Japanese study where it was identified in 36% of SSP/A [42, 43]. However, in serrated adenocarcinomas, the *BRAF* mutation, MSI and CIMP are all detected in more than 80% of samples [30] suggesting that high levels of MSI are more relevant in advanced stages of serrated tumorigenesis. Further genetic alterations which have been described in more advanced tumours include targets of MSI (*BAX, TGFßR2, IGFR2*, p53, often present late during adenoma/carcinoma progression), *CDKN2A*/*p16*, MINT genes, 3p, and 15q (CRAC1) [10, 33, 44]. Consistently, it was shown that the number of methylated genes increased significantly in the order of aberrant crypt foci (ACF) to SSA/P to serrated cancer [36].

However, beside of these few marker alterations, the molecular steps and predisposing genetic factors of sporadic and SPS-associated serrated lesions are largely unknown yet. To describe and characterise the mutation spectrum and pattern of serrated lesions, we have chosen to select a number of representative polyps present in a single 41 years old female patient with clinically confirmed SPS. This approach using a single patient is not sufficient to identify potential predisposing genetic factors of SPS (germline variants), however, it offers the opportunity to investigate a large number of polyps of different stages and colonic regions with the same constitutional genetic background. The recent identification of an SSP/A specific expression profile indicates that the underlying mechanisms are operating in both sporadic and syndromic SSP/As, so that it is likely that the results of SPS-associated polyps might be also representative for the sporadic counterpart [15].

The synchronus or metachronous occurrence of dozens of serrated polyps strongly argues for an underlying genetic basis, although it remains unclear so far, whether the predisposing genetic factors mainly act in a monogenic fashion, or contribute as low or moderately penetrant variants to a more complex, oligo/polygenic trait. Thus, it can be hypothesized that hyperplastic and serrated polyps originating from an SPS patient, share genetic features or involved pathways of tumorigenesis to a certain degree.

The aim of the present study was the systematic and exome-wide identification of point mutations (single base pair substitutions, small indels) in protein-coding genes as potential novel oncogenic drivers of serrated tumorigenesis rather than a comprehensive molecular approach covering all levels of possible alterations including methylation pattern and gross genomic alterations. By targeted and exome sequencing of eleven early hyperplastic / serrated polyps from all parts of the colon, we found a somatic mutational profile characteristic for SPS patients with the specific oncogenic *BRAF* and *KRAS* mutations present in more than half of the examined polyps which is comparable to other studies [45, 46]. It is known that the frequency of detected *BRAF* and *KRAS* mutations observed in SPS patients depends on the number and the location of polyps examined [45, 47].

Usually, SSP/As are larger than HPs and localized predominantly in the proximal colon [48, 49] which is similar to our findings. Interestingly, the specific *BRAF* or *KRAS* mutations were identified in almost all polyps from the proximal but no polyp from the distal colon although the relevant genomic regions had a minimum of 30× and 50× coverage for BRAF and KRAS, respectively, in the DNA derived from mutation negative polyps.

The association of proximal polyps with a *BRAF* mutation and *MLH1* methylation was already reported by others who suggested that this relation increases the risk of progression to malignancy [50].

All *BRAF* and *KRAS* mutations identified in the exome sequencing data of the polyps were confirmed by targeted molecular analysis which demonstrates the ability of our approach to identify relevant variants with a fraction of variant reads ^3^ 5% in tumour DNA.

Subsequently, all variants called in the exome sequencing data were filtered in multiple stringent steps to select for rare, non-polymorphic variants. No relevant variants were detected in 123 potential candidate genes for serrated tumorigenesis published in recent years including *RNF43*, several targets of CIMP, and MSI [19, 33]. Finally, 25 somatic variants, all of which were single base pair substitutions, were identified. Of these variants, 18 could not be validated by Sanger sequencing although the ratio of variant alleles in exome data was clearly over the detection threshold of Sanger sequencing. Interestingly, all of the non-validated variants were G > T transversions and in almost all of them the read depth was low, pointing to technical artifacts which is in line with previous observations in HiSeq exome data [51, 52].

Altogether, seven rare variants could be validated by Sanger sequencing in the examined polyps. They affect seven different genes and all are predicted to result in missense changes. Of note, five of the genes are related to functions relevant to tumour development such as proliferation, cell differentiation, or cell invasion. However, all variants occurred each in a single polyp only and none of the involved genes seem to belong to a shared pathway, i.e. there was no evidence for recurrently mutated genes or functionally related gene groups. Among the promising candidate genes, only the two variants of *ABI3BP* and *CATSPERB* are predicted to be deleterious. Thus, proof of the causal relevance of these variants remains challenging.

*ABI3BP (*also known as *TARSH, NESH, NESHBP)* is a positive regulator of cell-substrate adhesion and involved in an extracellular matrix organization. It plays an important role in proliferation and replicative senescence and may serve as a trigger of tumour development [53]. *ABI3BP* prevents chromosome instability in a p53-independent manner, resulting in suppression of carcinogenesis [54]. Down-regulation of *ABI3BP* expression has been found in thyroid tumorigenesis [55] and lung cancer cell lines [56], and thus, deleterious mutations of *ABI3AP* might contribute the tumour development.

*CATSPERB* is involved in cell differentiation. The *CATSPER* gene family is known to be relevant for spermatogenesis [57] and *CATSPER* SNPs have been reported to be associated with bone mineral density in premenopausal women [58], but there is no study which relates *CATSPER* variants to cancer so far.

Our results are consistent with the present knowledge regarding the pathology of early serrated polyps. There are only few published studies that aimed to identify genetic changes in early serrated polyps. Apart from *RNF43*, no convincing novel alterations were found across studies so far. In recent years, a number of candidate driver genes for SSP/A development and progression have been identified by expression analysis, however, the replication of these findings and the evaluation of the clinical and biological relevance has yet to be demonstrated. Our findings do not indicate that point mutations of these candidate genes are the underlying mechanism leading to aberrant expression. Recently, Sakai et al. performed a targeted mutation screening of 126 candidate cancer driver genes in 25 SSA/Ps and found somatic mutations in several cancer pathways [14]. However, although the SSA/Ps were larger (27 mm on average) compared to those examined in this study, they could not identify any mutation in 2, no mutation besides of *BRAF* in 9, and only one additional mutation besides of *BRAF* in 7 SSA/Ps. Therefore, the negative results of the present study compared to the study of Sakai et al. might indicate considerable genetic heterogeneity of somatic events in serrated polyps. In addition, the difference might be caused by the smaller size of polyps in the present study since less somatic events are expected to be present in early stages of tumorigenesis.

We cannot rule out that variants might have been overlooked due to a very low allelic fraction (tissue heterogeneity) or incomplete coverage. Moreover, we did not cover the whole spectrum of potential genetic and epigenetic alterations in serrated lesions since the analysis of variants outside the coding part of the genome (e.g. promoters, deep intronic regions, intergenic regions), aberrant methylation pattern, and structural rearrangements was beyond the scope of this study.

## Conclusions

To the best of our knowledge, this is the first study which performed exome sequencing in a number of serrated polyps from a single patient to identify potential novel drivers of serrated tumorigenesis. Our data indicate that somatic mutations beyond the well-known driver mutations in the established (*BRAF/KRAS*) and novel (*RNF43*) genes seem to be rare events in early *BRAF/KRAS-*related serrated lesions of SPS patients. No frequently affected genes and no enrichment of specific pathways have been observed. In addition, none of the current candidate genes known to be affected by epigenetic and/or expression changes harboured a relevant point mutation. Thus, other alterations such as specific epigenetic changes, in particular hypermethylation of tumour suppressor genes, or mutations in regulatory regions, which result in aberrant expression, might be the major driving force of tumour progression in sporadic and syndromic serrated polyps as proposed by other investigators [15, 32, 45]. This would be consistent with the recent observation that aberrant crypt foci (ACF) as the earliest precancerous lesions of the serrated pathway, arise by a *BRAF* mutation and methylation of a few genes and develop into SSP/As through accumulated methylation of a limited number of additional genes [15, 36].

## Additional files

Additional file 1: Table S1. Established causative or candidate genes for (hereditary) colorectal tumours and published genes related to serrated polyps (*n* = 74). **Table S2.** Primers used for validating 25 variants by Sanger sequencing. (PDF 239 kb)

Additional file 2: Figure S1. Sanger sequencing confirmed the *KRAS G12D* mutation in tumour T69 and the *BRAF* V600E mutation in tumour T71 and T76. (PDF 72 kb)

## Abbreviations

1°: The first degree
ACF: Aberrant crypt foci
BWA: Burrow-wheeler aligner
CIMP: CpG island methylator phenotype
CIN: Chromosomal instability
CRC: Colorectal cancer
DNA: Deoxyribonucleic acid
GATK: Genome Analysis Toolkit
HNPCC: Hereditary non polyposis colorectal cancer
HPS: Hyperplastic polyposis syndrome
HPs: Hyperplastic polyps
MAF: Minor allele frequency
MAP: *MUTYH*-associated polyposis
MAPK: Mitogen-activated protein kinase
mm: Millimeter
MSI: Microsatellite instability
MSS: Microsatellite stability
NCI: National Cancer Institute
NGS: Next generation sequencing
SAMtools: Sequence alignment/map tools
SNV: Single nucleotide variant
SPS: Serrated polyposis syndrome
SSP/A: Sessile serrated polyps/adenomas
TSA: Traditional serrated adenomas
WES: Whole exome sequencing
WHO: World Health Organization
