# Supplementary material for: Exome sequencing characterizes the somatic mutation spectrum of early serrated lesions in a patient with serrated polyposis syndrome (SPS)
Source: Hered Cancer Clin Pract. 2017 Nov 29;15:22. doi: 10.1186/s13053-017-0082-9 (PMC5707812; doi:10.1186/s13053-017-0082-9)
Supplement: Supplementary file 1 — Established causative or candidate genes for (hereditary) colorectal tumours and published genes related to serrated polyps (n = 74). Table S2. Primers used for validating 25 variants by Sanger sequencing. (PDF 239 kb) [file 13053_2017_82_MOESM1_ESM.pdf]

Supplementary Tables

Horpaopan et al.

Exome sequencing characterizes the somatic mutation spectrum of
early serrated lesions in a patient with serrated polyposis syndrome (SPS)

Supplementary Table S1 Established causative or candidate genes for (hereditary) colorectal tumours and published genes related to serrated polyps (n = 74)

No.	Gene	Chr	Disease / pathway / susceptibility	Ref.
1	*ACVR2*	2q22-23	Target gene in MMR-deficient CRC	[1]
2	*AHBD1*	2	Found LoF mutation in serrated polyposis families	[2]
3	*AIM2*	1q22	Target gene in MMR-deficient CRC	[1]
4	*APC*	5q22.2	Familial adenomatous polyposis (FAP)	[3]
5	*APC2*	19p13.3	Wnt signalling pathway, depletion of intracellular beta-catenin, specifically expressed in brain	[4]
6	*AXIN1*	16p13.3	Regulation of Wnt-beta-catenin signaling	[1]
7	*AXIN2*	17q24	Regulation of Wnt-beta-catenin signaling	[4, 5]
8	*BAX*	19q13.3	Target gene in MMR-deficient CRC	[1]
9	*BLM*	15q26.1	Target gene in MMR-deficient CRC	[1]
10	*BMPR1A*	10q22.3	Juvenile polyposis syndrome (JPS)	[4]
11	*BRAF*	7q34	Oncogene, somatic mutations in CRC	[6]
12	*BUB1B*	15q15	Gastrointestinal adenomas and carcinomas	[7]
13	*CA9*	9p13.3	Found LoF mutation in serrated polyposis families	[2]
14	*Caspase-5*	11q22.2	target gene in MMR-deficient CRC	[1]
15	*CASQ1*	1p3.2	Found LoF mutation in serrated polyposis families	[2]
16	*CDH1*	16q22.1	Hereditary diffuse gastric cancer (HDGC)	[8]
17	*CDX2*	13q12.3	target gene in MMR-deficient CRC	[1]
18	*CHEK2*	22q12.1	Susceptibility for CRC	[9, 10]
19	*CHK1*	11q22	Target gene in MMR-deficient CRC	[1]
20	*CNPY4*	7q22.1	Found LoF mutation in serrated polyposis families	[2]
21	*COL15A1*	9q22.3	Found LoF mutation in serrated polyposis families	[2]
22	*COL20A1*	20q13	Found LoF mutation in serrated polyposis families	[2]
23	*CREB3L3*	19p13	Found LoF mutation in serrated polyposis families	[2]
24	*CRYBA2*	2q35	Frequently overexpressed in *BRF*-mutant colon cancer	[11]
25	*CTNNB1*	3p21	canonical Wnt signaling pathway	[12]
26	*DDX31*	9q34	Found LoF mutation in serrated polyposis families	[2]
27	*E2F-4*	16q21	Target gene in MMR-deficient CRC	[1]
28	*EGFR*	7p12	Oncogene, somatic mutations in lung cancer	[13, 14]
29	*EMR2*	19p13	Found LoF mutation in serrated polyposis families	[2]
30	*ENDOG*	9q34.1	Found LoF mutation in serrated polyposis families	[2]
31	*EPCAM*	2p21	Lynch syndrome / HNPCC	[15]
32	*FAM123B*	Xq11.2	canonical Wnt signaling pathway	[16, 17]
33	*FBXW7*	4q31.3	Cyclin E degradation, frequently mutated in CRC	[4]
34	*FLI1*	11q24.3	Candidate serrated polyposis gene	[18]
35	*FSCN1*	7p22.1	Frequently overexpressed in *BRF*-mutant colon cancer	[11]
36	*FSIP2*	2q32.1	Found LoF mutation in serrated polyposis families	[2]

37	*GCA*	2q24.2	Found LoF mutation in serrated polyposis families	[2]
38	*GCSAM*	3q13.2	Found LoF mutation in serrated polyposis families	[2]
39	*GEM*	8q22	Found LoF mutation in serrated polyposis families	[2]
40	*GJA3*	13q12.11	Candidate serrated polyposis gene	[18]
41	*GPT*	8q24.3	Found LoF mutation in serrated polyposis families	[2]
42	*GREM1*	15q13.3	Hereditary mixed polyposis syndrome 1	[8]
43	*GRWD1*	19q13	Found LoF mutation in serrated polyposis families	[2]
44	*GSK3B*	3q13.3	Canonical Wnt signaling pathway	[5]
45	*H3F3B*	17q25	Found LoF mutation in serrated polyposis families	[2]
46	*HDAC2*	6q21	Candidate serrated polyposis gene	[19]
47	*HDHD3*	9q32	Candidate serrated polyposis gene	[18]
48	*hRAD50*	5q31	Target gene in MMR-deficient CRC	[1]
49	*IDO2*	8p11.21	Found LoF mutation in serrated polyposis families	[2]
50	*IGF2*	11p15.5	Candidate serrated polyposis gene	[19]
51	*IGFBP7*	4q12	Candidate serrated polyposis gene	[20]
52	*IGFIIR*	6q26-27	Target gene in MMR-deficient CRC	[1]
53	*IGSF6*	16p12.2	Found LoF mutation in serrated polyposis families	[2]
54	*IMPACT*	18q11	Found LoF mutation in serrated polyposis families	[2]
55	*KRAS*	12p12.1	Oncogene, frequently mutated in CRC	[6]
56	*MAP2K4*	17p12	MAPK pathway, mutated in CRC	[21, 22]
57	*MBD4*	3q21-q22	target gene in MMR-deficient CRC	[1]
58	*MGAT5B*	17q25	Found LoF mutation in serrated polyposis families	[2]
59	*MGMT*	10q26.3	Candidate serrated polyposis gene	[23]
60	*MLH1*	3p22.2	Candidate serrated polyposis gene	[20]
61	*MLH3*	14q24.3	Candidate serrated polyposis gene	[23]
62	*MSH2*	2p21	Candidate serrated polyposis gene	[23]
63	*MSH3*	5q11-q12	Target gene in MMR-deficient CRC	[1]
64	*MSH6*	2p16.3	Candidate serrated polyposis gene	[23]
65	*MUC6*	11p15	Frequently overexpressed in *BRF*-mutant colon cancer	[11]
66	*MUTYH*	1p32-34	*MUTYH*-associated polyposis (MAP)	[24]
67	*MYH15*	3q13.1	Found LoF mutation in serrated polyposis families	[2]
68	*NCAN*	19p13	Found LoF mutation in serrated polyposis families	[2]
69	*NRAS*	1p13.2	Noonan syndrome, rarely mutated in CRC	[25]
70	*OR4L1*	14q11	Found LoF mutation in serrated polyposis families	[2]
71	*PDGFRA*	4q12	Upregulated in CRC	[26]
72	*PECR*	2q35	Found LoF mutation in serrated polyposis families	[2]
73	*PIK3CA*	3q26.3	Cowden syndrome, frequently mutated in CRC	[4, 27]
74	*PMS2*	7p22.1	Candidate serrated polyposis gene	[23]
75	*POLD1*	19q13.3	Hereditary multiple adenomas / CRC	[28]
76	*POLD2*	7p13	Candidate for hereditary multiple adenomas / CRC	[28]
77	*POLD3*	11q14	Candidate for hereditary multiple adenomas / CRC	[28]
78	*POLD4*	11q13	Candidate for hereditary multiple adenomas / CRC	[28]
79	*POLE*	12q24.3	Hereditary multiple adenomas / CRC	[28]
80	*POLE2*	14q21	Candidate for hereditary multiple adenomas / CRC	[28]
81	*POLE3*	9q33	Candidate for hereditary multiple adenomas / CRC	[28]
82	*POLE4*	2p12	Candidate for hereditary multiple adenomas / CRC	[28]
83	*PPP2R1B*	11q23	MAPK pathway, candidate TSG, frequently mutated in CRC	[5]
84	*PQLC1*	18q23	Candidate serrated polyposis gene	[18]
85	*PTEN*	10q23.3	Target gene in MMR-deficient CRC	[1]
86	*PTPN13*	4q21	Found LoF mutation in serrated polyposis families	[2]
87	*PTPRJ*	11p11.2	Frequently mutated in colorectal adenomas / CRC	[8]

88	*PWP2*	21q22	Found LoF mutation in serrated polyposis families	[2]
89	*RASL10B*	17q12	Candidate serrated polyposis gene	[18]
90	*RGS19*	20q13	Found LoF mutation in serrated polyposis families	[2]
91	*RIZ*	1p36	Target gene in MMR-deficient CRC	[1]
92	*RNF43*	17q22	Play a role in serrated neoplasia pathway	[2]
93	*SEC16B*	1q25.2	Found LoF mutation in serrated polyposis families	[2]
94	*SCEL*	13q22.3	Found LoF mutation in serrated polyposis families	[2]
95	*SEC63*	6q21	Target gene in MMR-deficient CRC	[1]
96	*SEMG1*	20q13	Frequently overexpressed in *BRF*-mutant colon cancer	[11]
97	*SFRP1*	8p11.21	Candidate serrated polyposis gene	[19, 29]
98	*SLC22A2*	6q25.3	Found LoF mutation in serrated polyposis families	[2]
99	*SLC26A2*	5q32	Candidate serrated polyposis gene	[18]
100	*SLC27A6*	5q23.3	Found LoF mutation in serrated polyposis families	[2]
101	*SLC30A7*	1p21.2	Found LoF mutation in serrated polyposis families	[2]
102	*SLIT2*	4p15.31	Marker for CIMP	[23]
103	*SMAD2*	18q21.1	TGF-beta signaling pathway, frequently mutated in CRC	[4]
104	*SMAD4*	18q21.1	Juvenile polyposis syndrome (FJP)	[4]
105	*SSNA1*	9q34.3	Found LoF mutation in serrated polyposis families	[2]
106	*ST6GALNAC*	9q34.11	Found LoF mutation in serrated polyposis families	[2]
107	*STK11*	19p13.3	Target gene in MMR-deficient CRC	[1]
108	*SYTL1*	1p36.11	Found LoF mutation in serrated polyposis families	[2]
109	*TAS2R3*	7q34	Found LoF mutation in serrated polyposis families	[2]
110	*TBP*	6q27	Target gene in MMR-deficient CRC	[1]
111	*TCF4*	10q25.3	Target gene in MMR-deficient CRC	[1]
112	*TGFBR2*	3p22	Target gene in MMR-deficient CRC	[1]
113	*TP53*	17p13.1	Li-Fraumeni syndrome	[1]
114	*TRNP1*	1p36	Frequently overexpressed in *BRF*-mutant colon cancer	[11]
115	*USP45*	6q16.2	Found LoF mutation in serrated polyposis families	[2]
116	*WIF1*	12q14.3	Inhibitor of Wnt signaling pathway	[29]
117	*WRN*	8p12	Candidate serrated polyposis gene	[19]
118	*ZIC2*	13q32	Frequently overexpressed in *BRF*-mutant colon cancer	[11]
119	*ZIC5*	13q32	Frequently overexpressed in *BRF*-mutant colon cancer	[11]
120	*ZNF491*	19p13.2	Found LoF mutation in serrated polyposis families	[2]
121	*ZNF568*	19q13.12	Found LoF mutation in serrated polyposis families	[2]
122	*ZNF614*	19q13.41	Found LoF mutation in serrated polyposis families	[2]
123	*ZNF860*	3p23	Found LoF mutation in serrated polyposis families	[2]

CRC, colorectal cancer; HNPCC, hereditary nonpolyposis colorectal cancer; LoF, loss of function; MMR, mismatch repair

Supplementary Table S2 Primers used for validating 25 variants by Sanger sequencing

No.	Gene	Chr	Position	Ref	Allele	Mut cDNA	Protein	Tu-ID	Forward primer (5'-3')	Reverse primer (5'-3')	size
1	ABI3BP[#]	3	100499047	T	A	c.2036A>T	p.K679M	71	GTTTCAGAGCTGAGCTGTCA	CTGGCAACTCTACCAAGTTTC	196
2	CALD1[#]	7	134617870	G	A	c.332G>A	p.R111Q	69	AGTGGAAGGGGATGATGAGG	GAGCGACAGACTTGCATCTGT	136
3	CATSPERB[#]	14	92084032	G	T	c.2309C>A	p.P770H	71	TACATAACTGTCAGTATCATCAA	CCTTCAGAATAGATTCAGTTG	213
4	CCBP2[#]	3	42906178	A	T	c.184A>T	p.S62C	72	GGTGTCCTTTGGCAAAGTCT	GGCAGTGTCACCAGAAACAG	177
5	CENPQ	6	49459907	G	T	c.726G>T	p.L242F	79	GCGATTCGTTGACAATTATTG	GGTTGACATGCTCTTCATCTG	224
6	CNTN5	11	99942566	G	T	c.1429G>T	p.A477S	70	GCTGGAATGTATCAGTGTTTGG	AATTAGAGCATGAAAAGAAGCAAG	135
7	COL8A1[#]	3	99513118	G	A	c.373G>A	p.E125K	71	CATACCCCTTCTCTCTCTTCTCT	CCTTTAATTCCAGGTATCCCAT	144
8	CPZ	4	8621320	G	T	c.1935G>T	p.R645S	69, 71	CCACATTGTCATTGCCCAA	CTTTGTGGGATGTCTGTGGC	415
9	CSPG4	15	75968509	G	T	c.6351C>A	p.D2117E	69	CCACAGCTCCAGAGTGAGAC	CTAGATGCTGGCGAGCTGG	240
10	DNAI1[#]	9	34506717	G	A	c.1156G>A	p.V386I	71	AAGAACCCCAGCTTCCCTGA	TTGGCTGAGCTGCAGAAGGA	170
11	FBLN5	14	92349339	G	T	c.821C>A	p.P274H	69	ACAGCCTTACCTTGGCAGCTT	TCTCTGAGTTCCTCTGCCAACA	115
12	KIAA1033	12	105558425	G	T	c.3361G>T	p.E1121*	79	TTGGTATTTTGCCATAATAAGTA	TTAAACCACTCACTGACCTTC	177
13	KLHDC1	14	50201340	G	T	c.857G>T	p.C286F	78	ATTGTATCCCAGAGACCTAATCT	GAAAGATATCCTTCTCAATGGC	242
14	NEURL1B	5	172096909	G	T	c.153G>T	p.R51R	80	AGGAGTTCGGAGGAGGCCTAAC	ATCGTGCGCGGTGAAGCCGAA	330
15	NOL11	17	65716083	G	T	c.312+5G>T	SS	80	GTTGTACACGATAATAAGGTGAG	GAATGAAAACCTGATTAGGACCT	324
16	PPP3CB	10	75230956	G	T	c.681C>A	p.F227L	71	TCGGACCATAACAAGTCACAC	TTGGTATCGTTTTCCTATGCAC	138
17	PROB1	5	138728291	G	T	c.2480C>A	p.P827H	78	CGCTCCCTGGGAGGGGCTTT	TAGGGCCAGCAGGGGTCCGAT	239
18	PRR23A	3	138725070	G	T	c.41C>A	p.P14H	73	TGGCGGGAGCTCCAGCACCA	CGCTCCCTACGTCCTCTGCTA	296
19	RAPH1	2	204305718	G	T	c.2195C>A	p.P732Q	71	GCTGAACTTGATGGATCTTCAG	CAATGGAGTTGTTCCACCAC	146
20	RGS13	1	192613504	G	T	c.40G>T	p.D14Y	73	CCCAGTGACTGTATTCCATTAAC	CATATGACTGCTCTTCTGATGAA	365
21	RIF1	2	152321774	G	T	c.5740G>T	p.A1914S	78, 79	CCCCAAAAATGGAACTGAG	GGTTCTCTCTTGTCCATGAAAG	140
22	SLC27A3	1	153748613	G	T	c.781G>T	p.G261C	79	GTTTCTGTGGCTCTGGTTCG	GTTCGGAGCTCCAGCCTTA	151
23	TRIM13	13	50587208	G	T	c.1132G>T	p.G378W	69	TGGTCCTACCATGTTCCTAG	ACCAAATCTGAATTACCACTCT	308
24	UBE2CBP	6	83732195	G	T	c.823C>A	p.Q275K	80	GAGACCTTGGATTCCTAGGCT	CAGGTCTTGGTTTGTCCAGAG	162

25	VGLL2[#]	6	117589395	C	A	c.132C>A	p.S44R	70	ATCAGTCTGAACCGAATTTGC	TTATACTGGCTGGGGTTTGG	176

[#] mutations were validated

References

1. Grady WM, Carethers JM: **Genomic and epigenetic instability in colorectal cancer pathogenesis**. *Gastroenterology* 2008, **135**(4):1079-1099.

2. Yan HH, Lai JC, Ho SL, Leung WK, Law WL, Lee JF, Chan AK, Tsui WY, Chan AS, Lee BC *et al*: **RNF43 germline and somatic mutation in serrated neoplasia pathway and its association with BRAF mutation**. *Gut* 2016.

3. Groden J, Thliveris A, Samowitz W, Carlson M, Gelbert L, Albertsen H, Joslyn G, Stevens J, Spirio L, Robertson M *et al*: **Identification and characterization of the familial adenomatous polyposis coli gene**. *Cell* 1991, **66**(3):589-600.

4. Sjoblom T, Jones S, Wood LD, Parsons DW, Lin J, Barber TD, Mandelker D, Leary RJ, Ptak J, Silliman N *et al*: **The consensus coding sequences of human breast and colorectal cancers**. *Science* 2006, **314**(5797):268-274.

5. Suraweera N, Robinson J, Volikos E, Guenther T, Talbot I, Tomlinson I, Silver A: **Mutations within Wnt pathway genes in sporadic colorectal cancers and cell lines**. *Int J Cancer* 2006, **119**(8):1837-1842.

6. O'Brien MJ, Yang S, Mack C, Xu H, Huang CS, Mulcahy E, Amorosino M, Farraye FA: **Comparison of microsatellite instability, CpG island methylation phenotype, BRAF and KRAS status in serrated polyps and traditional adenomas indicates separate pathways to distinct colorectal carcinoma end points**. *Am J Surg Pathol* 2006, **30**(12):1491-1501.

7. de Voer RM, Geurts van Kessel A, Weren RD, Ligtenberg MJ, Smeets D, Fu L, Vreede L, Kamping EJ, Verwiel ET, Hahn MM *et al*: **Germline mutations in the spindle assembly checkpoint genes BUB1 and BUB3 are risk factors for colorectal cancer**. *Gastroenterology* 2013, **145**(3):544-547.

8. Venkatachalam R, Verwiel ET, Kamping EJ, Hoenselaar E, Gorgens H, Schackert HK, van Krieken JH, Ligtenberg MJ, Hoogerbrugge N, van Kessel AG *et al*: **Identification of candidate predisposing copy number variants in familial and early-onset colorectal cancer patients**. *Int J Cancer* 2011, **129**(7):1635-1642.

9. Suchy J, Cybulski C, Wokolorczyk D, Oszurek O, Gorski B, Debniak T, Jakubowska A, Gronwald J, Huzarski T, Byrski T *et al*: **CHEK2 mutations and HNPCC-related colorectal cancer**. *Int J Cancer* 2010, **126**(12):3005-3009.

10. Cybulski C, Wokolorczyk D, Kladny J, Kurzawski G, Suchy J, Grabowska E, Gronwald J, Huzarski T, Byrski T, Gorski B *et al*: **Germline CHEK2 mutations and colorectal cancer risk: different effects of a missense and truncating mutations?** *Eur J Hum Genet* 2007, **15**(2):237-241.

11. Kanth P, Bronner MP, Boucher KM, Burt RW, Neklason DW, Hagedorn CH, Delker DA: **Gene Signature in Sessile Serrated Polyps Identifies Colon Cancer Subtype**. *Cancer Prev Res (Phila)* 2016, **9**(6):456-465.

12. Pendas-Franco N, Aguilera O, Pereira F, Gonzalez-Sancho JM, Munoz A: **Vitamin D and Wnt/beta-catenin pathway in colon cancer: role and regulation of DICKKOPF genes**. *Anticancer Res* 2008, **28**(5A):2613-2623.

13. Pastorino U, Sozzi G, Miozzo M, Tagliabue E, Pilotti S, Pierotti MA: **Genetic changes in lung cancer**. *J Cell Biochem Suppl* 1993, **17F**:237-248.

14. Kim MJ, Lee EJ, Suh JP, Chun SM, Jang SJ, Kim DS, Lee DH, Lee SH, Youk EG: **Traditional serrated adenoma of the colorectum: clinicopathologic implications and endoscopic findings of the precursor lesions**. *Am J Clin Pathol* 2013, **140**(6):898-911.

15. Kuiper RP, Vissers LE, Venkatachalam R, Bodmer D, Hoenselaar E, Goossens M, Haufe A, Kamping E, Niessen RC, Hogervorst FB *et al*: **Recurrence and variability of germline EPCAM deletions in Lynch syndrome**. *Hum Mutat* 2011, **32**(4):407-414.

16. Sanz-Pamplona R, Lopez-Doriga A, Pare-Brunet L, Lazaro K, Bellido F, Alonso MH, Ausso S, Guino E, Beltran S, Castro-Giner F *et al*: **Exome Sequencing Reveals AMER1 as a Frequently Mutated Gene in Colorectal Cancer**. *Clin Cancer Res* 2015, **21**(20):4709-4718.

17. Major MB, Camp ND, Berndt JD, Yi X, Goldenberg SJ, Hubbert C, Biechele TL, Gingras AC, Zheng N, Maccoss MJ *et al*: **Wilms tumor suppressor WTX negatively regulates WNT/beta-catenin signaling**. *Science* 2007, **316**(5827):1043-1046.

18. Inoue M, Takahashi S, Soeda H, Shimodaira H, Watanabe M, Miura K, Sasaki I, Kato S, Ishioka C: **Gene-expression profiles correlate with the efficacy of anti-EGFR therapy and chemotherapy for colorectal cancer**. *Int J Clin Oncol* 2015, **20**(6):1147-1155.

19. Esteller M: **Epigenetics in cancer**. *N Engl J Med* 2008, **358**(11):1148-1159.

20. Kaji E, Uraoka T, Kato J, Hiraoka S, Suzuki H, Akita M, Saito S, Tanaka T, Ohara N, Yamamoto K: **Externalization of saw-tooth architecture in small serrated polyps implies the presence of methylation of IGFBP7**. *Dig Dis Sci* 2012, **57**(5):1261-1270.

21. Leary RJ, Lin JC, Cummins J, Boca S, Wood LD, Parsons DW, Jones S, Sjoblom T, Park BH, Parsons R *et al*: **Integrated analysis of homozygous deletions, focal amplifications, and sequence alterations in breast and colorectal cancers**. *Proc Natl Acad Sci U S A* 2008, **105**(42):16224-16229.

22. Lee JW, Soung YH, Kim SY, Nam SW, Park WS, Lee JY, Yoo NJ, Lee SH: **Kinase domain mutation of MAP2K4 is rare in gastric, colorectal and lung carcinomas**. *Pathology* 2006, **38**(3):263-264.

23. Beggs AD, Jones A, Shepherd N, Arnaout A, Finlayson C, Abulafi AM, Morton DG, Matthews GM, Hodgson SV, Tomlinson IP: **Loss of expression and promoter methylation of SLIT2 are associated with sessile serrated adenoma formation**. *PLoS Genet* 2013, **9**(5):e1003488.

24. Al-Tassan N, Chmiel NH, Maynard J, Fleming N, Livingston AL, Williams GT, Hodges AK, Davies DR, David SS, Sampson JR *et al*: **Inherited variants of MYH associated with somatic G:C-->T:A mutations in colorectal tumors**. *Nat Genet* 2002, **30**(2):227-232.

25. Irahara N, Baba Y, Nosho K, Shima K, Yan L, Dias-Santagata D, Iafrate AJ, Fuchs CS, Haigis KM, Ogino S: **NRAS mutations are rare in colorectal cancer**. *Diagn Mol Pathol* 2010, **19**(3):157-163.

26. Dai YC, Zhu XS, Nan QZ, Chen ZX, Xie JP, Fu YK, Lin YY, Lian QN, Sang QF, Zhan XJ: **Identification of differential gene expressions in colorectal cancer and polyp by cDNA microarray**. *World J Gastroenterol* 2012, **18**(6):570-575.

27. Nosho K, Kawasaki T, Ohnishi M, Suemoto Y, Kirkner GJ, Zepf D, Yan L, Longtine JA, Fuchs CS, Ogino S: **PIK3CA mutation in colorectal cancer: relationship with genetic and epigenetic alterations**. *Neoplasia* 2008, **10**(6):534-541.

28. Palles C, Cazier JB, Howarth KM, Domingo E, Jones AM, Broderick P, Kemp Z, Spain SL, Guarino E, Salguero I *et al*: **Germline mutations affecting the proofreading domains of POLE and POLD1 predispose to colorectal adenomas and carcinomas**. *Nat Genet* 2013, **45**(2):136-144.

29. Huang S, Zhong X, Gao J, Song R, Wu H, Zi S, Yang S, Du P, Cui L, Yang C *et al*: **Coexpression of SFRP1 and WIF1 as a prognostic predictor of favorable outcomes in patients with colorectal carcinoma**. *Biomed Res Int* 2014, **2014**:256723.
