# Supplementary material for: Exome sequencing characterizes the somatic mutation spectrum of early serrated lesions in a patient with serrated polyposis syndrome (SPS)
Source: Hered Cancer Clin Pract. 2017 Nov 29;15:22. doi: 10.1186/s13053-017-0082-9 (PMC5707812; doi:10.1186/s13053-017-0082-9)

**Supplementary Fig. S1** Sanger sequencing confirmed the *KRAS G12D* mutation in tumour T69 and the *BRAF* V600E mutation in tumour T71 and T76

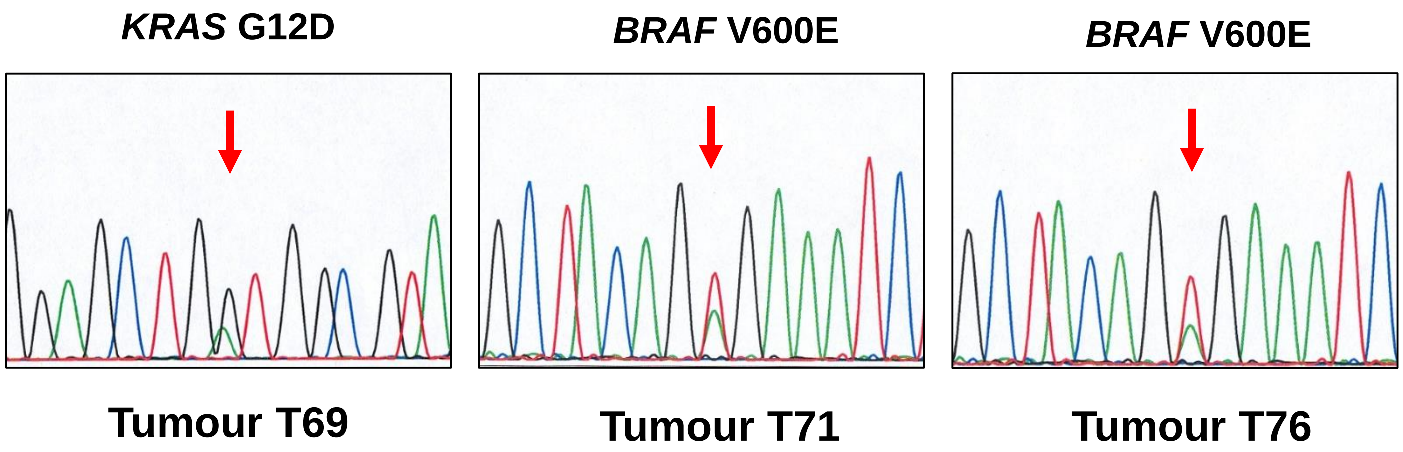

Supplement: Supplementary file 2 — Sanger sequencing confirmed the KRAS G12D mutation in tumour T69 and the BRAF V600E mutation in tumour T71 and T76. (PDF 72 kb) [file 13053_2017_82_MOESM2_ESM.pdf]
